# Molecular and Immune Features of Metastatic Prostate Cancer in Hispanic and Non‐Hispanic American Men

**DOI:** 10.1002/cnr2.70707

**Published:** 2026-10-25

**Authors:** Alejandro Recio‐Boiles, Sachin Kumar Deshmukh, Cassandra Jean Everly, Sharon Wu, Ricardo J. Estrada‐Mendizabal, Elisabeth Heath, Rana R. McKay, Cynthia Miranti, Ken Batai, Benjamin R. Lee, Juan Chipollini

**Affiliations:** ^1^ The University of Arizona Cancer Center Tucson Arizona USA; ^2^ Caris Life Sciences Phoenix Arizona USA; ^3^ Mayo Clinic Rochester Minnesota USA; ^4^ University of California San Diego California USA; ^5^ Roswell Park Comprehensive Cancer Center Buffalo New York USA

## Abstract

**Background:**

Prostate cancer (PCa) is the most common solid tumor in American men, with persistent ethnic disparities, particularly among Hispanic American (HA) men. The tumor microenvironment (TME) and tumor genomics may contribute to disease progression but remain undercharacterized in HA populations.

**Methods:**

We conducted a retrospective exploratory analysis of 83 patients with treatment‐naïve metastatic hormone‐sensitive prostate cancer who underwent next‐generation sequencing (NGS). Tumor genomic alterations, immune profiles, pathway activity, and clinical characteristics were compared between HA and non‐Hispanic White (NHW) patients.

**Results:**

Compared with NHW patients, HA patients presented with higher PSA levels (111.5 vs. 52.6 ng/mL, *p* = 0.02), and more advanced disease, including T4 tumors (41% vs. 14%, *p* = 0.01) and visceral metastases (41% vs. 16%, *p* = 0.04). Pathway analyses identified increased KRAS (FDR = 0.02), Hedgehog (FDR = 0.04), and NOTCH (FDR = 0.07) signaling in HA tumors. HA tumors exhibited higher expression of stem cell markers (*ALDH1A1*, *ALDH1A2*, *PROM1*) and immunoinhibitory genes (*PDCD1LG2*, *FOXP3*) (all *p* < 0.05), along with increased infiltration of M1 macrophages (0.72% vs. 0%) and natural killer (NK) cells (4.84% vs. 3.55%, all *p* < 0.05 and *q* < 0.10). HA tumors demonstrated higher expression of genes associated with drug efflux (*ABCC1, ABCB1*), EMT (*TWIST1, MMP2*), cell cycle (*CCND2*), apoptosis inhibition (*BIRC2, XIAP*), and epigenetic regulation (*EP300, TET1, TET2*) (all *p* < 0.05 and *q* < 0.10). Clinically, HA patients initiated therapy earlier (1 vs. 3 months, *p* ≤ 0.01), had a longer interval to second‐line therapy (20 vs. 11 months, *p* < 0.05), and had shorter follow‐up (23 vs. 44 months, *p* = 0.03).

**Conclusions:**

HA patients demonstrated differences in genomic alterations, pathway activity, and tumor immune features compared with NHW patients. Given the study's modest sample size and observational design, these findings should be considered hypothesis‐generating and warrant validation in larger prospective cohorts with broader representation of Hispanic American men.

## 1 Introduction

Prostate cancer (PCa) is the most common solid organ malignancy and the second leading cause of cancer‐related mortality among men in the United States [1, 2]. Racial and ethnic disparities in PCa outcomes are well documented. Black/African American men experience a disproportionately high burden of disease, with higher incidence and mortality rates than other racial groups, and have consequently been the focus of many studies investigating biological and socioeconomic contributors to PCa disparities [3]. Hispanic‐American (HA) men also experience disparities in presentation and outcomes compared with non‐Hispanic White (NHW) men, and remain substantially underrepresented in genomic and tumor microenvironment studies [4, 5, 6, 7].

While earlier studies emphasized social determinants of health as primary contributors to PCa disparities, accumulating evidence suggests that biological differences may also be associated with disease presentation and progression across racial and ethnic groups [8, 9]. Emerging evidence further suggests that epigenetic alterations, including differences in DNA methylation, chromatin remodeling, and gene expression regulation, may contribute to prostate cancer disparities by influencing tumor aggressiveness and treatment response across populations [10]. Advances in genomic profiling have identified molecular heterogeneity in prostate tumors that may correlate with differences in clinical behavior. However, most large‐scale genomic studies have predominantly included men of European ancestry, limiting the understanding of tumor biology in underrepresented populations, including HA men [11].

Emerging evidence from multiple cancer types suggests that racial and ethnic groups may exhibit distinct molecular tumor characteristics. Studies of hepatocellular, colorectal, gastric, lung, pancreatic, and central nervous system tumors have identified differences in genomic alterations, oncogenic pathway activation, and potentially actionable biomarkers among Hispanic patients and other underrepresented populations [12, 13, 14, 15, 16, 17]. Collectively, these findings highlight the importance of characterizing the molecular landscape of historically underrepresented populations to improve understanding of cancer biology and advance precision oncology.

The tumor microenvironment (TME), comprising stromal cells, immune cells, and vasculature, plays a critical role in tumor development, progression, and metastasis [18]. PCa is a biologically heterogeneous disease. Prostatic epithelial cells serve as the primary site of tumor development; however, prostate cancer progression is influenced not only by malignant epithelial cells but also by their interactions with surrounding stromal cells, immune cells, blood vessels, and extracellular matrix components. Reciprocal signaling among these cellular populations can promote tumor growth, invasion, metastasis, immune evasion, and therapeutic resistance, highlighting the importance of the tumor microenvironment in prostate cancer pathogenesis and progression [19]. Alterations in cytokine signaling, immune infiltration, and stromal remodeling have been shown to influence tumor growth and therapeutic response [20].

Recent studies have demonstrated that TME features differ across racial and ethnic groups and may influence PCa progression [19, 21]. Differences in immune response, stress response, cytokine signaling, and chemotaxis pathways have been observed in the TME of African American (AA) and NHW men, affecting fibroblast remodeling and angiogenesis [9, 21, 22, 23, 24]. These observations suggest that tumor‐associated stromal and immune responses differ across populations and may be associated with variation in disease presentation and outcomes. Although molecular and immune differences have been increasingly characterized in African American and non‐Hispanic White populations [1, 8, 9, 22, 24], comparable data in Hispanic American men remain limited. Consequently, the genomic, transcriptomic, and tumor microenvironment features of metastatic prostate cancer in this population remain poorly defined.

In this retrospective study, the primary objective was to characterize genomic, transcriptomic, and tumor microenvironment features of metastatic hormone‐sensitive prostate cancer in Hispanic American men compared with non‐Hispanic White men. Secondary exploratory objectives included evaluating differences in oncogenic signaling pathways, immune cell composition, and clinical characteristics between groups.

## 2 Methods

### 2.1 Patient Cohort and Clinical Data Collection

We conducted a retrospective analysis of 83 patients diagnosed with synchronous or metachronous metastatic treatment‐naive hormone‐sensitive prostate adenocarcinoma (mHSPC) who underwent next‐generation sequencing (NGS) of primary prostate gland tissue using the MI Profile‐MI Cancer Seek (Caris Life Sciences) and presented to the University of Arizona Cancer Center (UACC) between 2015 and 2023. For the purposes of this study, treatment‐naive refers to the absence of prior androgen deprivation therapy or other systemic hormonal therapy exposure at the time of tumor sampling. Primary tumor specimens were obtained before exposure to hormone‐directed therapy and were used for molecular profiling. Prior local therapies, including prostatectomy, radiation therapy, or neoadjuvant treatment, were permitted.

As the institution's Institutional Review Board (IRB) had provided a waiver of informed consent, informed consent was not obtained from patients included in this study. The IRB approval number was STUDY00000053. Patients were eligible for inclusion if they had histologically confirmed prostate adenocarcinoma, synchronous or metachronous metastatic or locally advanced hormone‐sensitive disease, available comprehensive Caris next‐generation sequencing (NGS) results, clinical documentation through at least first‐line hormone‐sensitive therapy, and documented self‐reported race and ethnicity in the medical record. Patients were excluded if they had a non‐predominant prostate cancer histology (including small cell, large cell neuroendocrine, squamous cell, adenoid cystic, stromal sarcoma, or other rare subtypes), localized disease managed with curative intent or active surveillance, unavailable or incomplete NGS data, insufficient clinical documentation to assess study outcomes, or missing race and ethnicity information.

Clinical and demographic data were abstracted from the electronic medical records in a de‐identified manner. Variables collected included age at presentation, race, ethnicity, obesity (defined as a body mass index ≥ 30 kg/m^2^), personal and family cancer history, Eastern Cooperative Oncology Group (ECOG) performance status, smoking status, alcohol use, and illicit drug use. Pathologic features at diagnosis included prostate‐specific antigen (PSA) level, Gleason grade, and tumor stage according to the American Joint Committee on Cancer (AJCC) 8th edition TNM classification. Treatment‐related variables included surgery, radiation, standard‐of‐care systemic treatment, and participation in clinical trials.

Clinical outcome data were collected to characterize treatment patterns and response kinetics. These included time from diagnosis to initiation of first‐line therapy, type of first‐line therapy administered, and time to PSA response following treatment initiation. Subsequent treatment patterns were recorded by assessing the interval from initiation of first‐line therapy to the initiation of second‐line treatment. Median overall survival was not reached at the time of analysis due to limited event occurrence and ongoing follow‐up for the majority of patients.

### 2.2 Next Generation Sequencing (NGS)

NGS was performed on genomic DNA isolated from formalin‐fixed paraffin‐embedded (FFPE) prostate cancer samples by a commercial CAP/CLIA lab using the NextSeq or NovaSeq 6000 platforms (Illumina Inc., San Diego, CA). For NextSeq sequenced tumors, a custom‐designed SureSelect XT assay was used to enrich 592 whole‐gene targets (Agilent Technologies, Santa Clara, CA). For NovaSeq sequenced tumors, a panel of more than 700 clinically relevant genes was sequenced at high coverage and high read‐depth, along with another panel designed to enrich for additional > 20 000 genes at a lower depth. All variants were detected with > 99% confidence based on allele frequency and amplicon coverage, with an average sequencing depth of coverage of > 500× and an analytic sensitivity of 5%. Prior to molecular testing, tumor enrichment was achieved by harvesting targeted tissue using manual microdissection techniques.

Genetic variants identified were interpreted by board‐certified molecular geneticists and categorized as “pathogenic,” “likely pathogenic,” “variant of unknown significance,” “likely benign,” or “benign” according to the American College of Medical Genetics and Genomics (ACMG) standards. Mutation assessment of individual genes stratified mutations as “pathogenic” and “likely pathogenic.” TMB was measured by counting all mutations found per tumor that had not been previously denoted as germline alterations in dbSNP151, Genome Aggregation Database (gnomAD) databases, or benign variants identified by Caris geneticists. A cutoff level of > 10 mutations per MB was used to characterize tumors as TMB high.

### 2.3 Fusion Detection

Fusions were analyzed using the ArcherDx FusionPlex Panel (ArcherDx, Boulder, CO), a target‐enrichment sequencing assay that utilizes Anchored Multiplex PCR with unidirectional gene‐specific primers to simultaneously detect and identify known and novel gene fusions. Libraries were created for use in NGS using ArcherDx reagents. After library preparation and indexing, samples were pooled and sequenced in a single reaction on the Illumina MiSeq system (Illumina, San Diego, CA). Sequencing data was de‐multiplexed and linked with the respective specimen by index for downstream fusion analysis using ArcherDx Analysis Software. All sequences were aligned to the human reference genome hg19. In order to qualify as “detected,” gene fusions were required to have ≥ 2 unique start sites and be in‐frame. Samples with < 20 000 unique RNA reads were reported as indeterminate and excluded from analysis. Off‐target hits and irrelevant novel isoforms were also excluded.

### 2.4 Whole Transcriptome Sequencing (WTS)

FFPE specimens underwent pathology review to diagnose percent tumor content and tumor size; a minimum of 10% of tumor content in the area for microdissection was required to enable enrichment and extraction of tumor‐specific RNA. A Qiagen RNA FFPE tissue extraction kit (Germantown, MD) was used for extraction, and the RNA quality and quantity were determined using the Agilent TapeStation (Santa Clara, CA). Biotinylated RNA baits were hybridized to the synthesized and purified cDNA targets, and the bait‐target complexes were amplified in a post capture PCR reaction. The resultant libraries were quantified, normalized, and the pooled libraries were denatured, diluted, and sequenced; the reference genome used was GRCh37/hg19 and analytical validation of this test demonstrated ≥ 97% Positive Percent Agreement (PPA), ≥ 99% Negative Percent Agreement (NPA), and ≥ 99% Overall Percent Agreement (OPA) with a validated comparator method. For transcript counting, transcripts per million (TPM) numbers were generated using the Salmon expression pipeline. RNA‐deconvolution was performed via quanTIseq to assess immune cell infiltration within the tumor microenvironment (TME) [25].

Epigenetic regulators evaluated included histone modifiers (*EZH2, EP300, BRD2, BRD3, BRD4, HDAC1, SIRT1, and KDM1A*) and DNA modifiers (*DNMT1, DNMT3A, TET1, TET2, and TET3*). Stem cell‐associated markers include *ALDH1A2, PROM1, CD44, ALDH1A3, ALDH1A1, SOX2, NANOG, KLF4, and POU5F1*.

T cell inflamed scores were calculated using T cell inflamed gene expression signature consisting of 160 genes, as previously described [26]. Interferon‐gamma (IFN‐γ) scores were calculated with a validated 18‐gene panel (CL5, CD27, CD274 (PD‐L1), CD276 (B7‐H3), CD8A, CMKLR1, CXCL9, CXCR6, HLA‐DQA1, HLA‐DRB1, HLA‐E, IDO1, LAG3, NKG7, PDCD1LG2 (PDL2), PSMB10, STAT1, and TIGIT) [27]. Gene set enrichment analysis (GSEA) was conducted to examine the enrichment or depletion of groups of genes associated with different biological pathways. This analysis was performed using Broad Institute software. GSEA was performed for biologically relevant pathways including KRAS, Hedgehog, NOTCH, WNT/β‐catenin, androgen response, PI3K/AKT/mTOR, epithelial‐to‐mesenchymal transition, apoptosis, hypoxia, angiogenesis, IL6/JAK/STAT3. IL2‐STAT5, p53, and TGF‐β signaling.

### 2.5 Immunohistochemistry

Immunohistochemistry of PD‐L1 (SP142 clone), MLH1 (M1 clone), MSH2 (G2191129 clone), MSH6 (44 clone), and PMS2 (EPR3947 clone) was completed on FFPE tissue slides. Slides were stained as per the manufacturer's instructions (Ventana Medical Systems Inc., Tucson, AZ) and were optimized and validated per CLIA/CAP and ISO requirements. Staining was scored for intensity (0 = no staining; 1+ = weak staining; 2+ = moderate staining; 3+ = strong staining) and staining percentage (0%–100%). The complete absence of protein expression of any of the 4 proteins tested (0+ in 100% of cells) was considered deficient MMR. A board‐certified pathologist independently evaluated all IHC results.

### 2.6 Statistical Analysis

Descriptive statistics were used to summarize clinical and molecular characteristics of the cohort. Continuous variables were compared using Student's *t*‐tests or Wilcoxon rank‐sum tests, as appropriate based on data distribution. Categorical variables were analyzed using chi‐square or Fisher's exact tests. Time‐to‐event outcomes were evaluated using the Kaplan–Meier method. Statistical significance was defined as a two‐sided *p*‐value < 0.05 and an FDR‐adjusted *q*‐value < 0.10. Given the exploratory nature of this study and the sample size, analyses were considered hypothesis‐generating.

## 3 Results

### 3.1 Clinical and Demographic Characteristics of the Study Cohort

This study cohort included 83 patients with mHSPC, of whom 32 self‐identified as HA. There was no difference between HA and NHW patients in the age at presentation (median 66 years), race (90% white), marital status (64% married), obesity (40% with body mass index > 30 kg/m^2^), personal cancer history (10%), or first‐degree family history of cancer (48%). HA patients had a higher frequency of smoking (69% vs. 54%) and alcohol or drug use (63% vs. 45%) compared to NHW patients (*p* = 0.04).

At presentation, a higher proportion of HA patients had an ECOG performance status of 0 compared with NHW patients (56% vs. 22%, *p* = 0.001). HA patients also presented with higher PSA levels (median 111.5 vs. 52.6 ng/mL, *p* = 0.02) and more advanced disease, including higher rates of T4 tumors (41% vs. 14%, *p* = 0.01) and visceral metastases (41% vs. 16%, *p* = 0.04). There were no significant differences between HA and NHW patients in the frequency of a Gleason score > 4 (79% vs. 73%, *p* = 0.61) or nodal involvement (N1: 69% vs. 61%, *p* = 0.85). Most patients presented with synchronous metastatic disease, while only two patients had metachronous metastatic progression. Owing to the limited number of metachronous cases, subgroup analyses based on timing of metastatic presentation were not performed. Additional demographic, clinical, and pathologic characteristics stratified by ethnicity are presented in Supplemental Table 1.

### 3.2 Comparative Genomic Landscape of HA and NHW Tumors

Tumors from HA patients demonstrated a higher frequency of TMPRSS2‐fusion compared with tumors from NHW patients (46.4% vs. 20.0%, *p* = 0.04) (Figure 1). Differences in the frequency of other genomic alterations, including *TP53*, SPOP, and CTNNB1 mutations, were observed but did not reach statistical significance. NHW tumors showed lower absolute frequencies of loss of heterozygosity (LOH), CDK12 alterations, and RB1 alterations compared to HA tumors, though these differences were not statistically significant.

**FIGURE 1:**
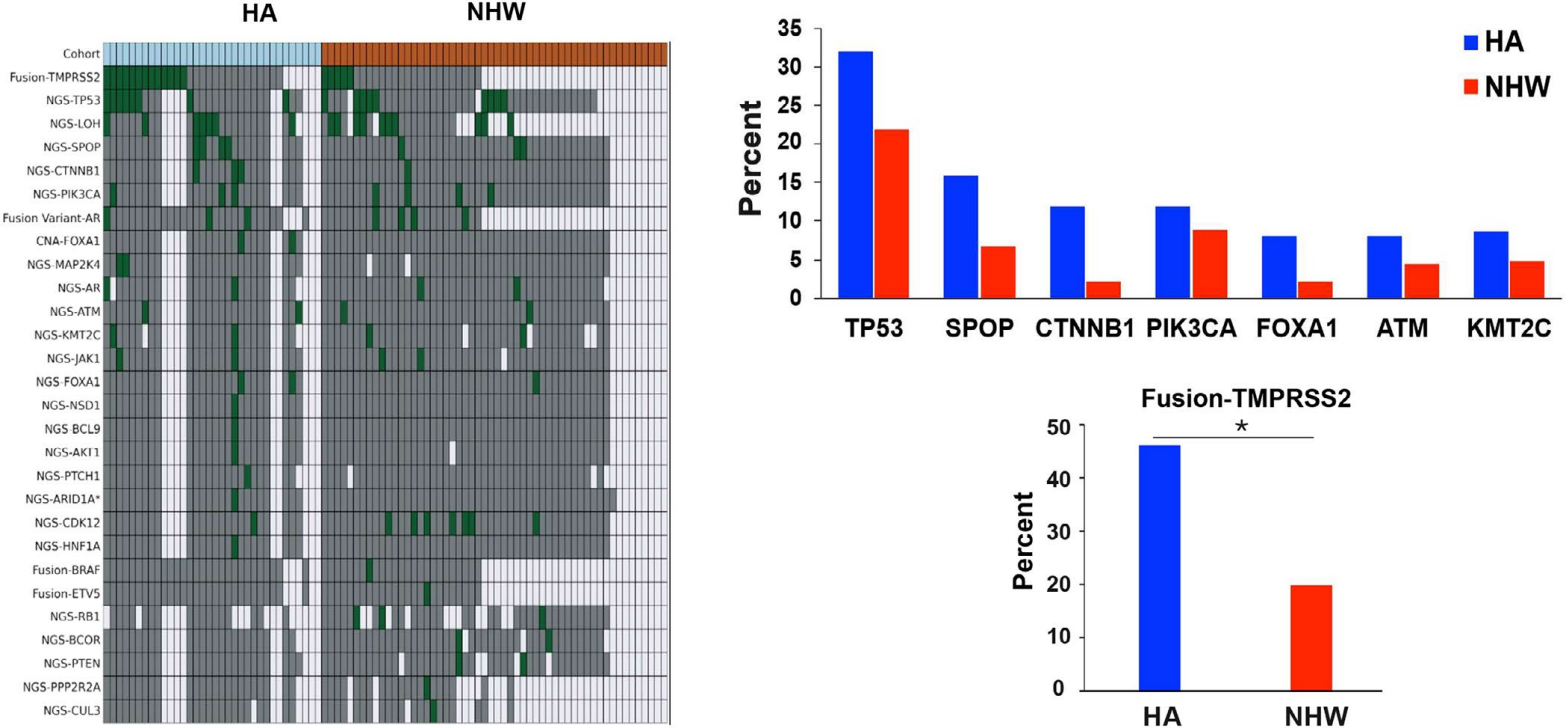
Comparative genomic alterations in metastatic hormone‐sensitive prostate cancer from Hispanic American and non‐Hispanic White patients. Genomic alterations identified by next‐generation sequencing (NGS) were compared between Hispanic American (HA; *n* = 32) and non‐Hispanic White (NHW; *n* = 51) patients with metastatic hormone‐sensitive prostate cancer. Left panel: Oncoprint summarizing the distribution of recurrent genomic alterations, copy number alterations (CNA), loss of heterozygosity (LOH), single nucleotide variants (SNVs), structural variants, and gene fusions across individual tumors. Each column represents one patient, and each row represents a genomic alteration. Colored boxes indicate the presence of an alteration, whereas gray boxes indicate no detected alteration. Upper right panel: Frequency of selected recurrent genomic alterations in HA and NHW tumors. Lower right panel: Frequency of TMPRSS2 gene fusions by ethnicity, demonstrating a significantly higher prevalence in HA tumors compared with NHW tumors (*p* = 0.04). Statistical comparisons were performed using the chi‐square or Fisher's exact test, as appropriate. CNA, copy number alteration; LOH, loss of heterozygosity; HA, Hispanic American; NGS, next‐generation sequencing; NHW, non‐Hispanic White; PCa, prostate cancer.

### 3.3 Tumor Immune Microenvironment Features

Immune deconvolution analysis demonstrated differences in immune cell composition between HA and NHW tumors. HA tumors showed higher infiltration of M1 Macrophages (0.72% vs. 0%, *p* < 0.05) and natural killer (NK) cells (4.84% vs. 3.55%, *p* < 0.05), along with lower infiltration of CD8 T cells (0% vs. 0.11%, *p* < 0.05) compared with NHW tumors (Figure 2). HA tumors also exhibited higher expression of the immunoinhibitory genes *PDCD1LG2* (1.68‐fold, *p* < 0.05) and *FOXP3* (1.45‐fold, *p* < 0.05). Moreover, there was no significant difference between HA and NHW in the T cell inflamed score (44 vs. −49, *p* = 0.14) and IFN‐y score (−0.5 vs. −0.45).

**FIGURE 2:**
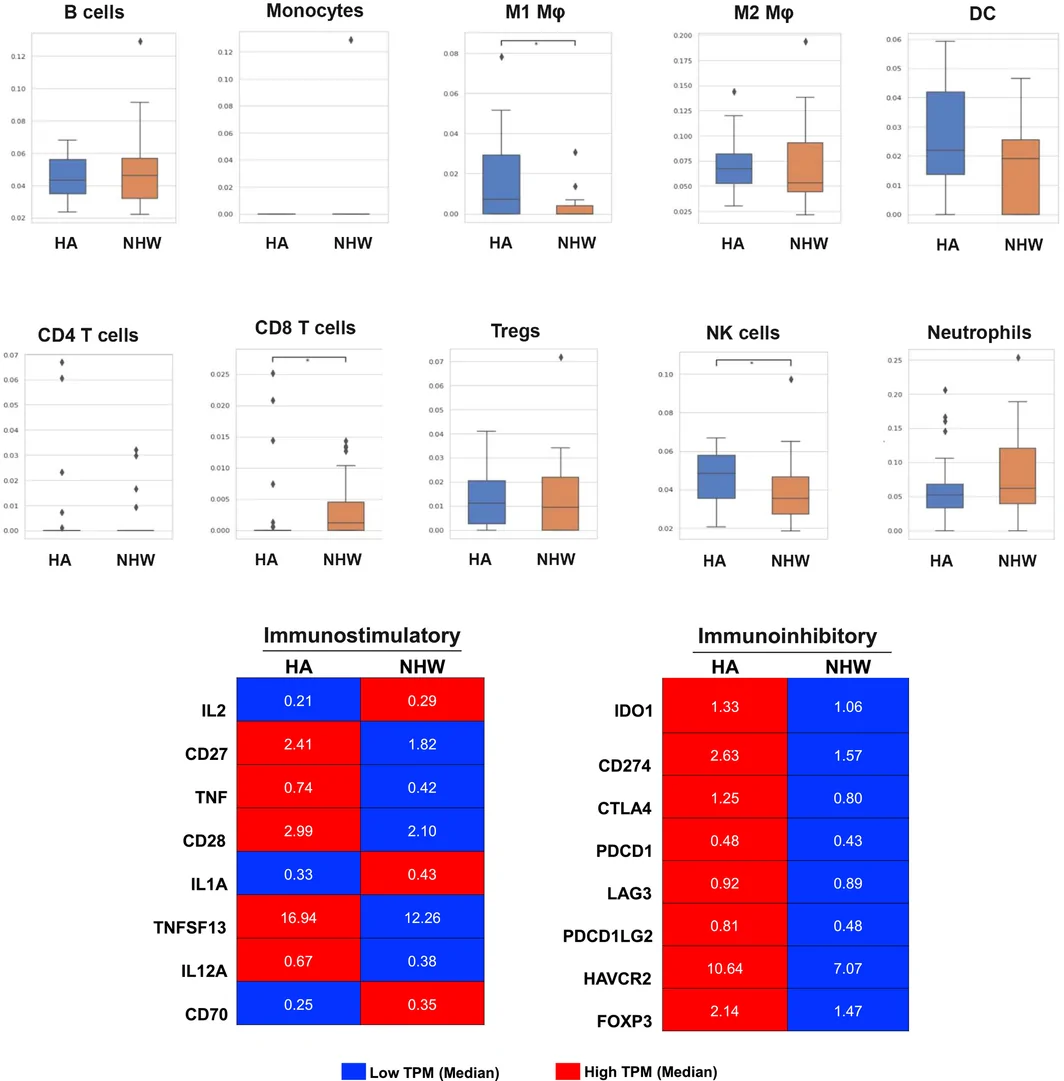
Immune cell infiltration and immune checkpoint gene expression in metastatic hormone‐sensitive prostate cancer from Hispanic American and non‐Hispanic White patients. Estimated immune cell composition was derived from whole‐transcriptome sequencing using quanTIseq and compared between Hispanic American (HA; *n* = 32) and non‐Hispanic White (NHW; *n* = 51) tumors. Left panel: Boxplots show the estimated median infiltration of major immune cell populations, including B cells, monocytes, M1 macrophages, M2 macrophages, dendritic cells (DC), CD4 T cells, CD8 T cells, regulatory T cells (Tregs), natural killer (NK) cells, and neutrophils. Boxes represent the interquartile range, the center line indicates the median, whiskers represent 1.5× the interquartile range, and points represent outliers. Right panel: Heatmaps display the median transcript expression (transcripts per million [TPM]) of representative immunostimulatory and immunoinhibitory genes in HA and NHW tumors. Red indicates higher median expression, and blue indicates lower median expression. Statistical comparisons between groups were performed using the Wilcoxon rank‐sum test. *p* < 0.05 was considered statistically significant and is indicated by an asterisk. DC, dendritic cells; HA, Hispanic American; NK, natural killer cells; NHW, non‐Hispanic White; TPM, transcripts per million; Tregs, regulatory T cells.

There were no significant differences between HA and NHW tumors in the frequency of PD‐L1 (10% vs. 2.1%, *p* = 0.29), mismatch repair deficiency or microsatellite instability high status (6.7% vs. 2.1%, *p* = 0.55), or high tumor mutational burden (4.0% vs. 4.5%, *p* = 1.0) (Supplemental Figure 1).

### 3.4 Pathway Enrichment Analysis

Gene set enrichment analysis demonstrated significant enrichment of Hedgehog signaling in HA tumors compared with NHW tumors (normalized enrichment score [NES]: 1.45, false discovery rate [FDR] = 0.04). Trends toward enrichment of NOTCH signaling (NES: 1.41, FDR = 0.07) and Wnt/β‐catenin signaling (NES: 1.38, FDR = 0.10) were also observed. Expression of pathway‐associated genes including *WNT2*, *WNT1, HES1*, and *NOTCH1* was higher in HA tumors compared with NHW tumors (all *p* < 0.05 and *q* < 0.10) (Figure 3).

**FIGURE 3:**
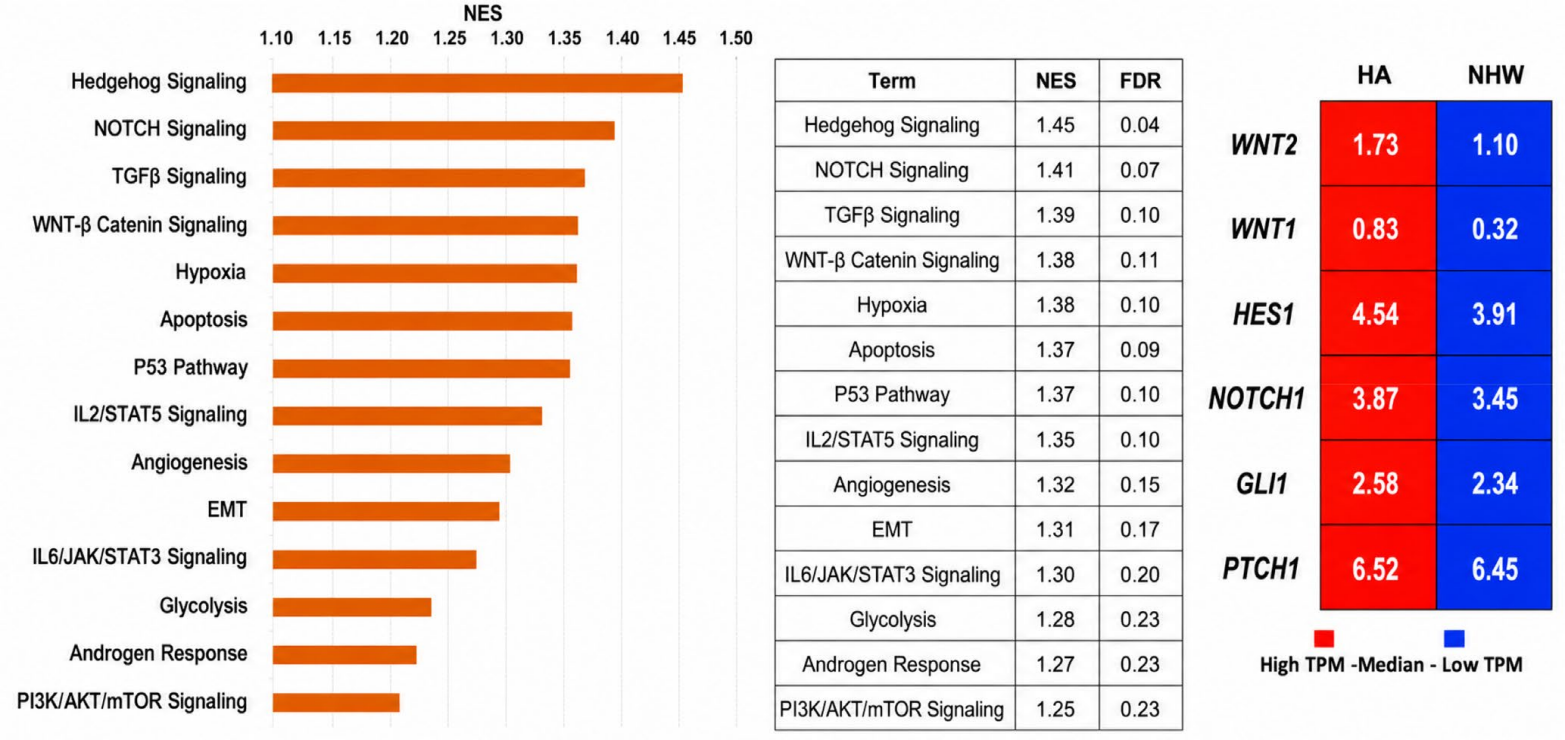
Gene set enrichment analysis of oncogenic signaling pathways in metastatic hormone‐sensitive prostate cancer. Gene set enrichment analysis comparing tumors from Hispanic American (HA; *n* = 32) and non‐Hispanic White (NHW; *n* = 51) patients. Bars represent normalized enrichment scores (NES), with higher positive values indicating greater pathway enrichment in HA tumors. The accompanying table lists the corresponding false discovery rate (FDR) for each pathway. The heatmap demonstrates median transcript expression (transcripts per million, TPM) of representative Hedgehog, NOTCH, and WNT/β‐catenin pathway genes, with red indicating higher expression and blue indicating lower expression.

### 3.5 Stem Cell‐Associated Gene Expression

HA tumors demonstrated higher expression of stem cell‐associated markers including *ALDH1A1* (2.1‐fold), *ALDH1A2* (3‐fold), and *PROM1* (1.6‐fold), compared with NHW tumors (all *p* < 0.05 and *q* < 0.10) (Figure 4).

**FIGURE 4:**
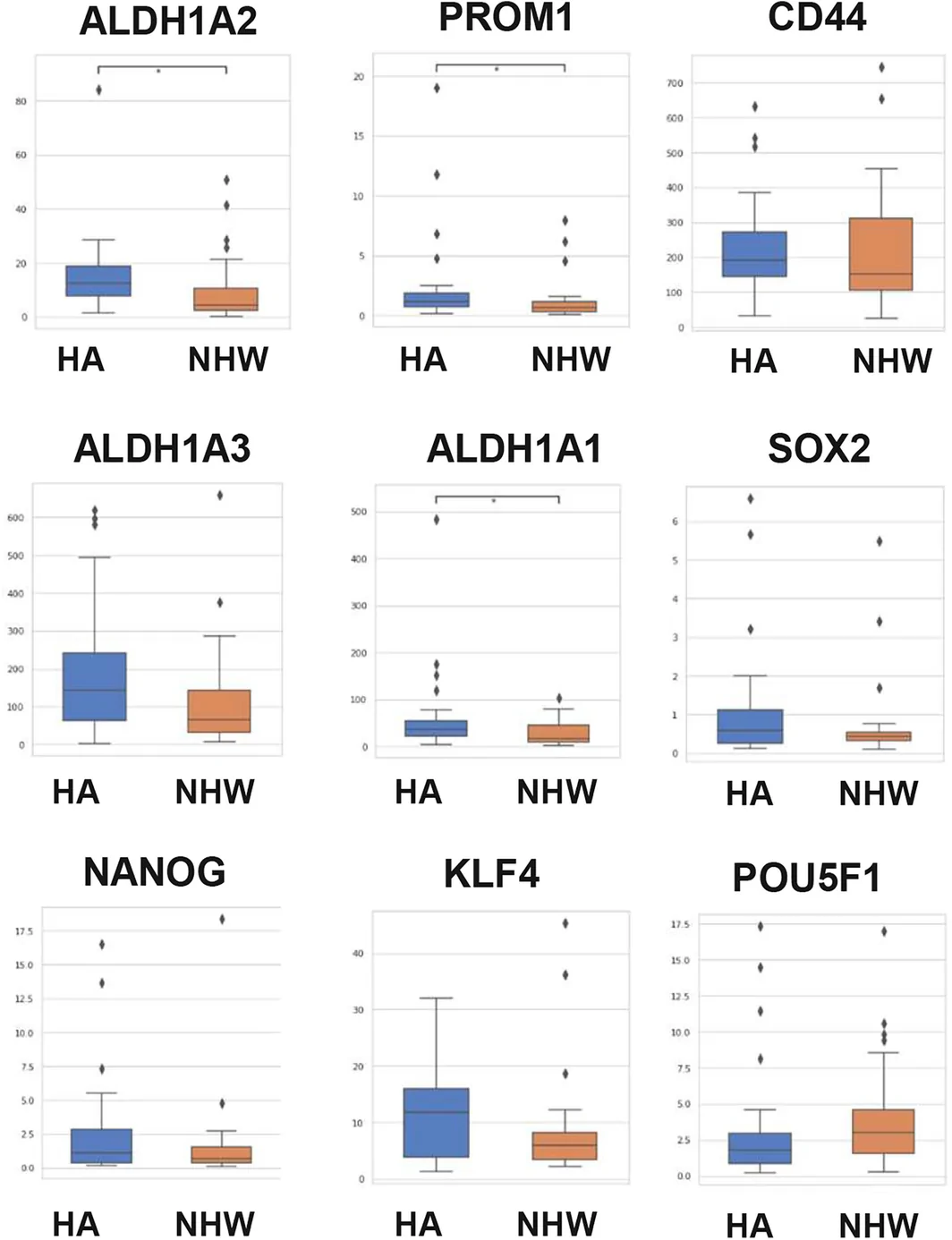
Expression of stemness‐associated genes in metastatic hormone‐sensitive prostate cancer from Hispanic American and non‐Hispanic White patients. Whole‐transcriptome sequencing was used to compare the expression of stemness‐associated genes between Hispanic American (HA; *n* = 32) and non‐Hispanic White (NHW; *n* = 51) tumors. Boxplots show median transcript expression (transcripts per million [TPM]) for ALDH1A2, PROM1 (CD133), CD44, ALDH1A3, ALDH1A1, SOX2, NANOG, KLF4, and POU5F1 (OCT4). These genes were selected because of their established roles in cancer stem cell biology, self‐renewal, and tumor initiation. Boxes represent the interquartile range, the center line indicates the median, whiskers extend to 1.5× the interquartile range, and points represent outliers. Statistical comparisons between groups were performed using the Wilcoxon rank‐sum test. *p* < 0.05 is indicated by an asterisk. HA, Hispanic American; NHW, non‐Hispanic White; TPM, transcripts per million.

### 3.6 Expression of Cancer Progression and Resistance Associated Genes

Compared with NHW tumors, HA tumors exhibited higher expression of genes associated with cell cycle regulation (*CCND2*), inhibition of apoptosis (*BIRC2*, *XIAP*), epithelial‐to‐mesenchymal transition (*TWIST1, MMP2*), and drug efflux (*ABCC1*, *ABCB1*), with fold changes ranging from 1.1 to 1.5 (all and *q* < 0.10) (Figure 5).

**FIGURE 5:**
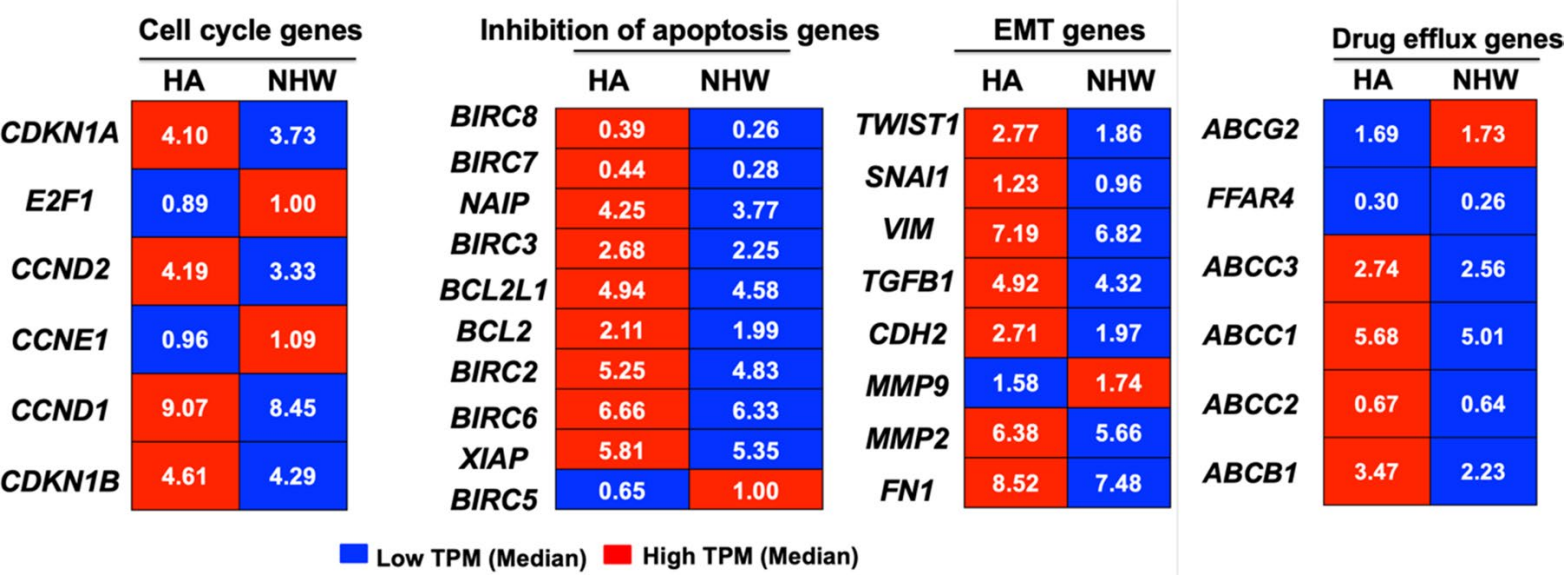
Expression of genes associated with cell cycle regulation, apoptosis, epithelial‐mesenchymal transition, and drug transport in metastatic hormone‐sensitive prostate cancer. Whole‐transcriptome sequencing was used to compare the expression of genes involved in biological processes associated with tumor progression between Hispanic American (HA; *n* = 32) and non‐Hispanic White (NHW; *n* = 51) tumors. Heatmaps display the median transcript expression (transcripts per million [TPM]) of representative genes involved in cell cycle regulation (CDKN1A, E2F1, CCND2, CCNE1, CCND1, CDKN1B), apoptosis regulation (BIRC8, BIRC7, NAIP, BIRC3, BCL2L1, BCL2, BIRC2, BIRC6, XIAP, BIRC5), epithelial–mesenchymal transition (EMT) (TWIST1, SNAI1, VIM, TGFB1, CDH2, MMP9, MMP2, FN1), and drug transport/efflux (ABCG2, FFAR4, ABCC3, ABCC1, ABCC2, ABCB1). Red indicates higher median gene expression, and blue indicates lower median gene expression. These genes were selected based on their established roles in tumor proliferation, apoptosis resistance, epithelial plasticity, and multidrug resistance. Statistical comparisons between groups were performed using the Wilcoxon rank‐sum test, and genes shown were significantly different between groups (*p* < 0.05 and *q* < 0.10). HA, Hispanic American; NHW, non‐Hispanic White; TPM, transcripts per million.

### 3.7 Treatment Patterns and Clinical Outcomes

Treatment modalities and sequencing did not differ significantly between HA and NHW patients (Supplemental Table 2). The median time from diagnosis to initiation of first‐line therapy was shorter among HA patients compared with NHW patients (1 vs. 3 months, *p* < 0.01) (Supplemental Figure 2A). Combination therapy was administered as first‐line treatment in 84% of all patients. Abiraterone plus androgen deprivation therapy (ADT) was used more frequently as first‐line therapy in HA patients compared with NHW patients (47% vs. 20%, *p* = 0.06), whereas ADT plus any AR blocker was more common in NHW patients (41% vs. 28%, *p* = 0.06).

Median PSA levels at three months following treatment initiation were lower in HA patients than in NHW patients (0.8 vs. 3.96 ng/mL, *p* = 0.05) (Supplemental Table 2). The median time to initiation of second‐line therapy was longer for HA patients compared with NHW patients (20 vs. 11 months, *p* = 0.05) (Supplemental Figure 2B). Median time to last clinical contact was shorter in HA patients than in NHW patients (23 vs. 44 months, *p* = 0.03) (Supplemental Figure 2C). Detailed treatment characteristics and outcomes by ethnicity are provided in Supplemental Table 2.

## 4 Discussion

In this retrospective clinicogenomic analysis of metastatic hormone‐sensitive prostate cancer, we identified differences in genomic alterations, pathway activity, and tumor immune features between HA and NHW cohorts. These findings provide a descriptive characterization of the molecular landscape in an underrepresented population and highlight several pathways and immune features that warrant further investigation. Our findings demonstrate that HA patients in this cohort presented with more advanced clinical disease and had differences in several molecular and immune features compared with NHW men. Specifically, HA patients had higher PSA levels, more advanced T and M stage disease, a higher frequency of TMPRSS2 gene fusions, increased expression of immunoinhibitory genes, and increased infiltration of M1 macrophages and NK cells. Additionally, HA tumors showed enrichment of oncogenic signaling pathways, stemness‐associated gene expression, and genes related to epithelial‐mesenchymal transition, cell cycle regulation, apoptosis resistance, drug efflux, and epigenetic modification. Together, these features reflect molecular patterns associated with aggressive tumor behavior in prior studies; however, the present analysis cannot establish their relationship to the more advanced clinical presentation observed in the HA cohort.

E26 Transformation‐Specific (ETS) gene family fusions were commonly observed in the HA cohort, including ERG, ETV1, and ETV4 rearrangements, with TMPRSS2 and SLC45A3 serving as fusion partners. The TMPRSS2‐ERG fusion is among the most frequent somatic alterations in prostate cancer, and it is thought to occur early in tumorigenesis [28]. Prior studies have demonstrated variability in the prevalence and mechanisms of ETS fusions across racial and ethnic groups, with the highest rates reported in men of European ancestry and lower rates in men of African and Asian ancestry [29]. However, the prevalence of ETS fusions in HA men has not been well described. Experimental data suggest that inflammatory signaling and oxidative stress may contribute to the formation of TMPRSS2–ERG rearrangements, raising the possibility that differences in inflammatory tumor environments may be associated with fusion frequency in specific populations [30]. The present study was not designed to evaluate the mechanisms underlying differences in fusion frequency. These observations should be considered hypothesis‐generating and require validation in independent cohorts.

Analysis of the tumor immune microenvironment revealed higher infiltration of M1 macrophages and natural killer (NK) cells, alongside lower CD8+ T‐cell infiltration, in HA tumors compared with NHW tumors. While M1 macrophages and NK cells are generally associated with anti‐tumor immune responses, persistent innate immune activation without effective adaptive immunity has been linked to tumor‐promoting inflammation and immune dysregulation in solid tumors [31, 32]. NK cells provide rapid cytotoxic responses, particularly against tumors with reduced major histocompatibility complex class I expression [33, 34]. However, tumors may adapt through immunosuppressive signaling, potentially limiting durable immune‐mediated control.

Consistent with the observed differences in immune cell composition, HA tumors demonstrated increased expression of immunoinhibitory markers, including PDCD1LG2 and FOXP3. A tumor microenvironment dominated by innate immune cells without sustained CD8+ T‐cell engagement may be associated with immune suppression over time [35]. These findings identify differences in immune composition and signaling between the study groups, although their relationship to tumor behavior and clinical outcomes remains uncertain. This study cannot determine whether these immune differences reflect ethnicity‐associated biology, differences in disease burden, or other clinical factors.

Pathway‐level analyses further demonstrated enrichment of Hedgehog signaling in HA tumors, with trends toward increased NOTCH and WNT/β‐catenin pathway activity. We observed upregulation of pathway‐associated genes, including WNT1, WNT2, HES1, and NOTCH1. These pathways have been implicated in prostate cancer stemness, EMT, and resistance to androgen receptor‐directed therapies in prior studies [36, 37, 38]. In addition, activation of WNT/β‐catenin signaling has been associated with T‐cell exclusion from the tumor microenvironment in prior studies [39, 40]. Although these previously described biological functions provide context for our observations, the present study cannot establish that pathway enrichment contributed to immune composition, treatment response, or clinical presentation. These findings instead identify candidate pathways for further investigation in HA prostate cancer.

HA tumors exhibited higher expression of cancer stem cell markers, including ALDH1A1, ALDH1A2, and PROM1, as well as genes associated with proliferation (CCND2), apoptosis resistance (BIRC2, XIAP), EMT (TWIST1, MMP2), and drug efflux (ABCC1, ABCB1). These molecular features have been associated with aggressive tumor behavior and therapeutic resistance in prior studies across cancer types.

Despite these molecular differences, HA and NHW patients received comparable systemic therapies. HA patients initiated first‐line therapy earlier and demonstrated more favorable initial PSA responses, yet had shorter follow‐up and evidence of more rapid progression. These findings suggest that while HA tumors may initially respond to androgen deprivation, underlying tumor biology may limit response durability.

HA patients had a longer interval to initiation of second‐line therapy than NHW patients, which may reflect sustained initial disease control or delayed recognition of progression. The retrospective design, selection bias related to NGS availability, incomplete clinical documentation, limited longitudinal outcome data, and shorter follow‐up among HA patients limit interpretation of these clinical outcomes. The modest sample size and potential confounders further constrain conclusions.

In addition, several baseline clinical characteristics differed between Hispanic American and non‐Hispanic White patients, including performance status, disease stage, visceral metastatic burden, smoking and substance use history, and duration of follow‐up. Given the modest sample size and exploratory nature of this study, multivariable analyses adjusting for these factors were not feasible. Furthermore, the limited sample size relative to the number of molecular and immune features evaluated would have increased the risk of model overfitting and unstable estimates. Consequently, the observed molecular and immune differences should be interpreted as unadjusted associations and may reflect, in part, differences in disease burden, clinical presentation, tissue selection, or other unmeasured factors rather than ethnicity alone. Accordingly, causal relationships between ethnicity, the observed molecular features, and clinical outcomes cannot be inferred. These findings should therefore be considered hypothesis‐generating and require validation in larger cohorts with sufficient power for multivariable analyses.

Strengths of this study include integrated genomic and immune profiling in a real‐world metastatic prostate cancer cohort and a focus on an underrepresented population. However, prospective studies with larger, ethnically diverse cohorts and longer follow‐up are needed to validate these findings. Overall, our results underscore the importance of incorporating Hispanic American men into clinicogenomic research and identify molecular and immune features that warrant further investigation in this population. These data support further investigation into biologically informed strategies to improve outcomes in high‐risk populations.

## 5 Conclusion

Our integrated molecular profiling of mHSPC identified differences in genomic alterations, pathway enrichment, and tumor immune features between Hispanic American and non‐Hispanic White patients. Hispanic American men demonstrated a higher prevalence of potentially actionable molecular features and a more advanced clinical presentation, findings that may be consistent with differences in tumor biology and disease characteristics. Given the retrospective design, modest sample size, and potential for residual confounding, these observations should be considered hypothesis‐generating. Validation in larger, multi‐institutional cohorts will be important to confirm these findings and to better understand the molecular features of prostate cancer in historically underrepresented populations.

## Author Contributions

**Alejandro Recio‐Boiles:** conceptualization, methodology, formal analysis, writing – original draft, writing – review and editing. **Sachin Kumar Deshmukh:** methodology, formal analysis, data curation, writing – review and editing. **Cassandra Jean Everly:** formal analysis, writing – original draft, writing – review and editing. **Sharon Wu:** data curation, formal analysis, writing – review and editing. **Ricardo J. Estrada‐Mendizabal:** data curation, formal analysis, writing – original draft, writing – review and editing. **Elisabeth Heath:** writing – review and editing, supervision. **Rana R. McKay:** writing – review and editing, supervision. **Cynthia Miranti:** methodology, formal analysis, writing – review and editing. **Ken Batai:** conceptualization, formal analysis, writing – review and editing, methodology. **Benjamin R. Lee:** writing – review and editing, supervision. **Juan Chipollini:** conceptualization, formal analysis, writing – review and editing, methodology.

## Funding

2P30 CA023074 supplement of the Cancer Center Support Grant from the NCI/NIH to the University of Arizona Cancer Center. Department of Urology Seed Grant from the Dr. George W Drach Endowment at the University of Arizona. The Biostatistics and Bioinformatics Shared Resource (BBSR) at the University of Arizona Cancer Center (UACC) provided statistical design, accurate data management, appropriate data analyses, and data interpretation.

## Ethics Statement

This study was approved by the University of Arizona Institutional Review Board (IRB). All procedures were conducted in accordance with the institution's ethical standards and the 1964 Declaration of Helsinki and its subsequent amendments.

## Consent

IRB provided a waiver of written informed consent for all participants.

## Conflicts of Interest

Dr. Rana R. McKay has served consulting or advisory role for the following companies: Pfizer, Janssen, Novartis, Tempus, Pfizer, Astellas Medivation, Dendreon, Bayer, Sanofi, Vividion, Calithera, Caris Life Sciences, Sorrento Therapeutics, AVEO, Seattle Genetics, Telix, Eli Lilly, Blue Earth Diagnostics, Ambrx, Sumitomo Pharma Oncology, Esiai, NeoMorph, Arcus Biosciences, Daiichi Sankyo, Exelixis, Bristol Myers Squibb, Merck, AstraZeneca, and Myovant.

## Supporting information


**Supplementary Figure 1** Comparison of clinically relevant immunotherapy biomarkers between Hispanic American and non‐Hispanic White patients with metastatic hormone‐sensitive prostate cancer. Immunohistochemical and genomic biomarkers associated with immunotherapy response were compared between Hispanic American (HA; *n* = 32) and non‐Hispanic White (NHW; *n* = 51) patients with metastatic hormone‐sensitive prostate cancer. Upper panel: Frequency of programmed death‐ligand 1 (PD‐L1) expression, mismatch repair deficiency/high microsatellite instability (dMMR/MSI‐H), and high tumor mutational burden (TMB ≥ 10 mutations/Mb) by ethnicity. PD‐L1 expression was assessed by immunohistochemistry using the SP142 antibody clone. dMMR was defined as complete loss of expression of one or more mismatch repair proteins (MLH1, MSH2, MSH6, or PMS2) by immunohistochemistry. TMB was determined by next‐generation sequencing and categorized as high using a threshold of ≥ 10 mutations/Mb. Lower panel: Number and percentage of positive cases in each cohort with corresponding *p* values. Statistical comparisons between groups were performed using Fisher's exact test. No statistically significant differences were observed between HA and NHW tumors for PD‐L1 expression (*p* = 0.29), dMMR/MSI‐H status (*p* = 0.55), or TMB‐high status (*p* = 1.00). dMMR, mismatch repair deficiency; HA, Hispanic American; Mb, megabase; MSI‐H, microsatellite instability‐high; NHW, non‐Hispanic White; PD‐L1, programmed death‐ligand 1; TMB, tumor mutational burden.

**Supplementary Figure 2A** Time from diagnosis to initiation of first‐line systemic therapy by ethnicity. Kaplan–Meier curves comparing the time from diagnosis to initiation of first‐line systemic therapy between Hispanic American (HA; *n* = 32) and non‐Hispanic White (NHW; *n* = 51) patients with metastatic hormone‐sensitive prostate cancer. Patients who had not initiated first‐line therapy at the time of last follow‐up were censored. Median time to first‐line treatment was **1.0 month** (95% confidence interval [CI], 0.5–2.0) for HA patients and **3.0 months** (95% CI, 2.0–9.0) for NHW patients. Survival curves were estimated using the Kaplan–Meier method and compared using the log‐rank test (*p* = 0.0016). CI, confidence interval; HA, Hispanic American; NHW, non‐Hispanic White.

**Supplementary Figure 2B** Time from initiation of first‐line therapy to initiation of second‐line therapy by ethnicity. Kaplan–Meier curves comparing the time from initiation of first‐line systemic therapy to initiation of second‐line therapy between Hispanic American (HA; *n* = 15) and non‐Hispanic White (NHW; *n* = 40) patients with metastatic hormone‐sensitive prostate cancer who received second‐line treatment. Patients who had not initiated second‐line therapy by the time of last follow‐up were censored. The median time from first‐line to second‐line therapy was **20.0 months** (95% confidence interval [CI], 8.0–23.0) for HA patients and **11.0 months** (95% CI, 9.0–13.0) for NHW patients. Survival curves were estimated using the Kaplan–Meier method and compared using the log‐rank test (*p* = 0.0544). CI, confidence interval; HA, Hispanic American; NHW, non‐Hispanic White.

**Supplementary Figure 2C** Time to last clinical contact by ethnicity. Kaplan–Meier curves comparing the time from diagnosis to last documented clinical follow‐up between Hispanic American (HA; *n* = 32) and non‐Hispanic White (NHW; *n* = 51) patients with metastatic hormone‐sensitive prostate cancer. Time to last contact was used to characterize the duration of clinical follow‐up for each cohort. Median follow‐up was **23.0 months** (95% confidence interval [CI], 11.0–36.0) for HA patients and **44.0 months** (95% CI, 32.0–58.0) for NHW patients. Curves were estimated using the Kaplan–Meier method and compared using the log‐rank test (*p* = 0.0267). CI, confidence interval; HA, Hispanic American; NHW, non‐Hispanic White.

**Supplementary Table 1** Baseline demographic, clinical, and pathologic characteristics of Hispanic American and non‐Hispanic White patients with metastatic hormone‐sensitive prostate cancer. Baseline demographic, clinical, and pathologic characteristics were compared between Hispanic American (HA; *n* = 32) and non‐Hispanic White (NHW; *n* = 51) patients with metastatic hormone‐sensitive prostate cancer. Variables include patient demographics, preferred language, body mass index (BMI), personal and family cancer history, smoking and alcohol/drug use, Eastern Cooperative Oncology Group (ECOG) performance status, Gleason score and Grade Group, and TNM stage at diagnosis. Continuous variables are presented as median (interquartile range), and categorical variables are presented as number (%). Comparisons between groups were performed using the Wilcoxon rank‐sum test for continuous variables and the chi‐square or Fisher's exact test for categorical variables. *p* values < 0.05 were considered statistically significant. BMI, body mass index; ECOG, Eastern Cooperative Oncology Group performance status; HA, Hispanic American; NA, data not available; NHW, non‐Hispanic White; TNM, Tumor‐Node‐Metastasis staging system (8th edition).

**Supplementary Table 2** Clinical management, treatment characteristics, and outcomes of Hispanic American and non‐Hispanic White patients with metastatic hormone‐sensitive prostate cancer. Clinical treatment characteristics and outcomes were compared between Hispanic American (HA; *n* = 32) and non‐Hispanic White (NHW; *n* = 51) patients with metastatic hormone‐sensitive prostate cancer. Variables include receipt of local therapies (radical prostatectomy and definitive radiation), neoadjuvant therapy, participation in clinical trials, hormonal therapy, prostate‐specific antigen (PSA) measurements, and treatment timing. Continuous variables are presented as median (interquartile range) or median (95% confidence interval), as appropriate, and categorical variables are presented as number (%). Comparisons between groups were performed using the Wilcoxon rank‐sum test for continuous variables and the chi‐square or Fisher's exact test for categorical variables. *p* values < 0.05 were considered statistically significant. ADT, androgen deprivation therapy; CI, confidence interval; Dx, diagnosis; HA, Hispanic American; NA, not applicable; NHW, non‐Hispanic White; PSA, prostate‐specific antigen.

## Data Availability

The authors elect not to share data.

## References

[1] K. D. Miller , A. P. Ortiz , P. S. Pinheiro , et al., “Cancer Statistics for the US Hispanic/Latino Population, 2021,” CA: A Cancer Journal for Clinicians 71, no. 6 (2021): 466–487, 10.3322/caac.21695. 34545941

[2] N. Hinata and M. Fujisawa , “Racial Differences in Prostate Cancer Characteristics and Cancer‐Specific Mortality: An Overview,” World Journal of Mens Health 40, no. 2 (2022): 217–227, 10.5534/wjmh.210070. PMC8987139 35021294

[3] J. W. Lillard , K. A. Moses , B. A. Mahal , and D. J. George , “Racial Disparities in Black Men With Prostate Cancer: A Literature Review,” Cancer 128, no. 21 (2022): 3787–3795, 10.1002/cncr.34433. PMC9826514 36066378

[4] R. J. Estrada‐Mendizabal , A. S. Dhaliwal , A. J. Bertolo , et al., “Prostate Cancer Disparities in Metastatic and Treatment Status for Hispanic Americans Based on Country of Origin Compared to Non‐Hispanic Whites Using the National Cancer Database,” Clinical Genitourinary Cancer 22, no. 1 (2024): e148–e155.e1, 10.1016/j.clgc.2023.10.002. 37903669

[5] D. Y. Lichtensztajn , J. T. Leppert , J. D. Brooks , et al., “Undertreatment of High‐Risk Localized Prostate Cancer in the California Latino Population,” Journal of the National Comprehensive Cancer Network : JNCCN 16, no. 11 (2018): 1353–1360, 10.6004/jnccn.2018.7060. PMC6314834 30442735

[6] F. M. Chinea , V. N. Patel , D. Kwon , et al., “Ethnic Heterogeneity and Prostate Cancer Mortality in Hispanic/Latino Men: A Population‐Based Study,” Oncotarget 8, no. 41 (2017): 69709–69721, 10.18632/oncotarget.19068. PMC5642510 29050235

[7] T. R. Rebbeck , “Prostate Cancer Disparities by Race and Ethnicity: From Nucleotide to Neighborhood,” Cold Spring Harbor Perspectives in Medicine 8, no. 9 (2018): a030387, 10.1101/cshperspect.a030387. PMC6120694 29229666

[8] D. Lowder , K. Rizwan , C. McColl , et al., “Racial Disparities in Prostate Cancer: A Complex Interplay Between Socioeconomic Inequities and Genomics,” Cancer Letters 531 (2022): 71–82, 10.1016/j.canlet.2022.01.028. PMC9701576 35122875

[9] R. R. Reams , D. Agrawal , M. B. Davis , et al., “Microarray Comparison of Prostate Tumor Gene Expression in African‐American and Caucasian American Males: A Pilot Project Study,” Infect Agent Cancer 4, no. Suppl 1 (2009): S3, 10.1186/1750-9378-4-S1-S3. PMC2638462 19208208

[10] J. B. Kaushal , P. Raut , S. Muniyan , et al., “Racial Disparity in Prostate Cancer: An Outlook in Genetic and Molecular Landscape,” Cancer Metastasis Reviews 43, no. 4 (2024): 1233–1255, 10.1007/s10555-024-10193-8. PMC11560487 38902476

[11] N. Weise , J. Shaya , J. Javier‐Desloges , H. H. Cheng , L. Madlensky , and R. R. McKay , “Disparities in Germline Testing Among Racial Minorities With Prostate Cancer,” Prostate Cancer and Prostatic Diseases 25, no. 3 (2022): 403–410, 10.1038/s41391-021-00469-3. PMC8590439 34775478

[12] D. Das , X. Wang , Y. C. Chiu , et al., “Integrative Multi‐Omics Characterization of Hepatocellular Carcinoma in Hispanic Patients,” Journal of the National Cancer Institute 116, no. 12 (2024): 1961–1978, 10.1093/jnci/djae207. PMC11630563 39189979

[13] D. M. Hein , W. Deng , M. Bleile , et al., “Racial and Ethnic Differences in Genomic Profiling of Early Onset Colorectal Cancer,” Journal of the National Cancer Institute 114, no. 5 (2022): 775–778, 10.1093/jnci/djac014. PMC9086761 35134211

[14] M. B. Mirza , J. Choi , P. Marincola Smith , et al., “ERBB2 Amplification in Gastric Cancer: A Genomic Insight Into Ethnic Disparities,” Journal of the National Cancer Institute 116, no. 11 (2024): 1830–1833, 10.1093/jnci/djae147. PMC11542984 38991830

[15] J. K. Tabor , A. Dincer , J. O'Brien , et al., “Variations in the Genomic Profiles and Clinical Behavior of Meningioma by Racial and Ethnic Group,” Journal of Neurosurgery 141, no. 3 (2024): 664–672, 10.3171/2024.1.JNS231633. 38518289

[16] J. Carrot‐Zhang , G. Soca‐Chafre , N. Patterson , et al., “Genetic Ancestry Contributes to Somatic Mutations in Lung Cancers From Admixed Latin American Populations,” Cancer Discovery 11, no. 3 (2021): 591–598, 10.1158/2159-8290.CD-20-1165. PMC7933062 33268447

[17] C. Monge , B. Waldrup , F. G. Carranza , S. Manjarrez , and E. Velazquez‐Villarreal , “Pathway‐Specific Genomic Alterations in Pancreatic Cancer Across Populations at Risk,” International Journal of Molecular Sciences 26, no. 16 (2025): 7695, 10.3390/ijms26167695. PMC12386847 40869017

[18] N. M. Anderson and M. C. Simon , “The Tumor Microenvironment,” Current Biology 30, no. 16 (2020): R921–R925, 10.1016/j.cub.2020.06.081. PMC8194051 32810447

[19] S. Goswami , C. Sarkar , S. Singh , A. P. Singh , and D. Chakroborty , “Racial Differences in Prostate Tumor Microenvironment: Implications for Disparate Clinical Outcomes and Potential Opportunities,” Cancer Health Disparities 6 (2022): 214. PMC9910060 36777283

[20] J. K. Messex and G. Y. Liou , “Impact of Immune Cells in the Tumor Microenvironment of Prostate Cancer Metastasis,” Life 13, no. 2 (2023): 333, 10.3390/life13020333. PMC9967893 36836690

[21] M. Gillard , R. Javier , Y. Ji , et al., “Elevation of Stromal‐Derived Mediators of Inflammation Promote Prostate Cancer Progression in African‐American Men,” Cancer Research 78, no. 21 (2018): 6134–6145, 10.1158/0008-5472.CAN-17-3810. 30181178

[22] T. A. Wallace , R. L. Prueitt , M. Yi , et al., “Tumor Immunobiological Differences in Prostate Cancer Between African‐American and European‐American Men,” Cancer Research 68, no. 3 (2008): 927–936, 10.1158/0008-5472.CAN-07-2608. 18245496

[23] M. A. Kinseth , Z. Jia , F. Rahmatpanah , et al., “Expression Differences Between African American and Caucasian Prostate Cancer Tissue Reveals That Stroma Is the Site of Aggressive Changes,” International Journal of Cancer 134, no. 1 (2014): 81–91, 10.1002/ijc.28326. PMC3800217 23754304

[24] J. Yuan , K. H. Kensler , Z. Hu , et al., “Integrative Comparison of the Genomic and Transcriptomic Landscape Between Prostate Cancer Patients of Predominantly African or European Genetic Ancestry,” PLoS Genetics 16, no. 2 (2020): e1008641, 10.1371/journal.pgen.1008641. PMC7046294 32059012

[25] F. Finotello , C. Mayer , C. Plattner , et al., “Molecular and Pharmacological Modulators of the Tumor Immune Contexture Revealed by Deconvolution of RNA‐Seq Data,” Genome Medicine 11, no. 1 (2019): 34, 10.1186/s13073-019-0638-6. PMC6534875 31126321

[26] R. Bao , D. Stapor , and J. J. Luke , “Molecular Correlates and Therapeutic Targets in T Cell‐Inflamed Versus Non‐T Cell‐Inflamed Tumors Across Cancer Types,” Genome Medicine 12, no. 1 (2020): 90, 10.1186/s13073-020-00787-6. PMC7590690 33106165

[27] R. Cristescu , R. Mogg , M. Ayers , et al., “Pan‐Tumor Genomic Biomarkers for PD‐1 Checkpoint Blockade‐Based Immunotherapy,” Science 362, no. 6411 (2018): eaar3593, 10.1126/science.aar3593. PMC6718162 30309915

[28] K. H. Stopsack , X. A. Su , J. B. Vaselkiv , et al., “Transcriptomes of Prostate Cancer With TMPRSS2:ERG and Other ETS Fusions,” Molecular Cancer Research 21, no. 1 (2023): 14–23, 10.1158/1541-7786.MCR-22-0446. PMC9812892 36125519

[29] C. K. Zhou , D. Young , E. D. Yeboah , et al., “TMPRSS2:ERG Gene Fusions in Prostate Cancer of West African Men and a Meta‐Analysis of Racial Differences,” American Journal of Epidemiology 186, no. 12 (2017): 1352–1361, 10.1093/aje/kwx235. PMC5860576 28633309

[30] R. S. Mani , M. A. Amin , X. Li , et al., “Inflammation‐Induced Oxidative Stress Mediates Gene Fusion Formation in Prostate Cancer,” Cell Reports 17, no. 10 (2016): 2620–2631, 10.1016/j.celrep.2016.11.019. PMC5147555 27926866

[31] A. Mantovani , S. Sozzani , M. Locati , P. Allavena , and A. Sica , “Macrophage Polarization: Tumor‐Associated Macrophages as a Paradigm for Polarized M2 Mononuclear Phagocytes,” Trends in Immunology 23, no. 11 (2002): 549–555, 10.1016/s1471-4906(02)02302-5. 12401408

[32] F. R. Greten and S. I. Grivennikov , “Inflammation and Cancer: Triggers, Mechanisms, and Consequences,” Immunity 51, no. 1 (2019): 27–41, 10.1016/j.immuni.2019.06.025. PMC6831096 31315034

[33] M. D. Vesely , M. H. Kershaw , R. D. Schreiber , and M. J. Smyth , “Natural Innate and Adaptive Immunity to Cancer,” Annual Review of Immunology 29 (2011): 235–271, 10.1146/annurev-immunol-031210-101324. 21219185

[34] E. Vivier , E. Tomasello , M. Baratin , T. Walzer , and S. Ugolini , “Functions of Natural Killer Cells,” Nature Immunology 9, no. 5 (2008): 503–510, 10.1038/ni1582. 18425107

[35] M. Binnewies , E. W. Roberts , K. Kersten , et al., “Understanding the Tumor Immune Microenvironment (TIME) for Effective Therapy,” Nature Medicine 24, no. 5 (2018): 541–550, 10.1038/s41591-018-0014-x. PMC5998822 29686425

[36] L. Marignol , K. Rivera‐Figueroa , T. Lynch , and D. Hollywood , “Hypoxia, Notch Signalling, and Prostate Cancer,” Nature Reviews. Urology 10, no. 7 (2013): 405–413, 10.1038/nrurol.2013.110. PMC5240418 23712204

[37] N. Takebe , L. Miele , P. J. Harris , et al., “Targeting Notch, Hedgehog, and Wnt Pathways in Cancer Stem Cells: Clinical Update,” Nature Reviews. Clinical Oncology 12, no. 8 (2015): 445–464, 10.1038/nrclinonc.2015.61. PMC4520755 25850553

[38] V. Kumar , M. Vashishta , L. Kong , et al., “The Role of Notch, Hedgehog, and Wnt Signaling Pathways in the Resistance of Tumors to Anticancer Therapies,” Frontiers in Cell and Development Biology 9 (2021): 9, 10.3389/fcell.2021.650772. PMC8100510 33968932

[39] J. J. Luke , R. Bao , R. Sweis , S. Spranger , and T. F. Gajewski , “WNT/β‐Catenin Pathway Activation Correlates With Immune Exclusion Across Human Cancers,” Clinical Cancer Research: An Official Journal of the American Association for Cancer Research 25, no. 10 (2019): 3074–3083, 10.1158/1078-0432.CCR-18-1942. PMC6522301 30635339

[40] X. Li , Y. Xiang , F. Li , C. Yin , B. Li , and X. Ke , “WNT/β‐Catenin Signaling Pathway Regulating T Cell‐Inflammation in the Tumor Microenvironment,” Frontiers in Immunology 10 (2019): 10, 10.3389/fimmu.2019.02293. PMC6775198 31616443

