# Supplementary material for: Molecular and Immune Features of Metastatic Prostate Cancer in Hispanic and Non‐Hispanic American Men
Source: Cancer Rep (Hoboken). 2026 Oct 25;9(10):e70707. doi: 10.1002/cnr2.70707 (PMC13696747; doi:10.1002/cnr2.70707)
Supplement: Supplementary file 1 [file CNR2-9-e70707-s001.docx]

Supplementary Figure 1. **Comparison of clinically relevant immunotherapy biomarkers between Hispanic American and non-Hispanic White patients with metastatic hormone-sensitive prostate cancer.** Immunohistochemical and genomic biomarkers associated with immunotherapy response were compared between Hispanic American (HA; n = 32) and non-Hispanic White (NHW; n = 51) patients with metastatic hormone-sensitive prostate cancer. Upper panel: Frequency of programmed death-ligand 1 (PD-L1) expression, mismatch repair deficiency/high microsatellite instability (dMMR/MSI-H), and high tumor mutational burden (TMB ≥10 mutations/Mb) by ethnicity. PD-L1 expression was assessed by immunohistochemistry using the SP142 antibody clone. dMMR was defined as complete loss of expression of one or more mismatch repair proteins (MLH1, MSH2, MSH6, or PMS2) by immunohistochemistry. TMB was determined by next-generation sequencing and categorized as high using a threshold of ≥10 mutations/Mb. Lower panel: Number and percentage of positive cases in each cohort with corresponding p values. Statistical comparisons between groups were performed using Fisher's exact test. No statistically significant differences were observed between HA and NHW tumors for PD-L1 expression (p = 0.29), dMMR/MSI-H status (p = 0.55), or TMB-high status (p = 1.00). Abbreviations: HA, Hispanic American; NHW, non-Hispanic White; PD-L1, programmed death-ligand 1; dMMR, mismatch repair deficiency; MSI-H, microsatellite instability-high; TMB, tumor mutational burden; Mb, megabase.
