# Supplementary material for: Molecular and Immune Features of Metastatic Prostate Cancer in Hispanic and Non‐Hispanic American Men
Source: Cancer Rep (Hoboken). 2026 Oct 25;9(10):e70707. doi: 10.1002/cnr2.70707 (PMC13696747; doi:10.1002/cnr2.70707)
Supplement: Supplementary file 2 [file CNR2-9-e70707-s002.docx]

**Supplementary Figure 2C. Time to last clinical contact by ethnicity.** Kaplan-Meier curves comparing the time from diagnosis to last documented clinical follow-up between Hispanic American (HA; n = 32) and non-Hispanic White (NHW; n = 51) patients with metastatic hormone-sensitive prostate cancer. Time to last contact was used to characterize the duration of clinical follow-up for each cohort. Median follow-up was **23.0 months** (95% confidence interval [CI], 11.0-36.0) for HA patients and **44.0 months** (95% CI, 32.0-58.0) for NHW patients. Curves were estimated using the Kaplan-Meier method and compared using the log-rank test (p = 0.0267). **Abbreviations:** HA, Hispanic American; NHW, non-Hispanic White; CI, confidence interval.

Log Rank Test=0.0267

| Time to Last Contact by Ethnicity | | | | | |
| --- | --- | --- | --- | --- | --- |
|  | N | Median Time to Last Contact | Std. Error | 95% Confidence Interval | |
| NHW | 51 | 44.0 | 4.6 | 32.0 | 58.0 |
| HA | 32 | 23.0 | 6.4 | 11.0 | 36.0 |
