# Supplementary material for: Molecular and Immune Features of Metastatic Prostate Cancer in Hispanic and Non‐Hispanic American Men
Source: Cancer Rep (Hoboken). 2026 Oct 25;9(10):e70707. doi: 10.1002/cnr2.70707 (PMC13696747; doi:10.1002/cnr2.70707)
Supplement: Supplementary file 3 [file CNR2-9-e70707-s004.docx]

**Supplementary Figure 2A. Time from diagnosis to initiation of first-line systemic therapy by ethnicity.** Kaplan–Meier curves comparing the time from diagnosis to initiation of first-line systemic therapy between Hispanic American (HA; n = 32) and non-Hispanic White (NHW; n = 51) patients with metastatic hormone-sensitive prostate cancer. Patients who had not initiated first-line therapy at the time of last follow-up were censored. Median time to first-line treatment was **1.0 month** (95% confidence interval [CI], 0.5–2.0) for HA patients and **3.0 months** (95% CI, 2.0–9.0) for NHW patients. Survival curves were estimated using the Kaplan–Meier method and compared using the log-rank test (p = 0.0016). **Abbreviations:** HA, Hispanic American; NHW, non-Hispanic White; CI, confidence interval.

Log Rank Test=0.0016

| Time to First Line of Treatment by Ethnicity | | | | | |
| --- | --- | --- | --- | --- | --- |
|  | N | Median Time to First Line | Std. Error | 95% Confidence Interval | |
| NHW | 51 | 3.0 | 1.1 | 2.0 | 9.0 |
| HA | 32 | 1.0 | 0.2 | 0.5 | 2.0 |
