# Supplementary material for: Molecular and Immune Features of Metastatic Prostate Cancer in Hispanic and Non‐Hispanic American Men
Source: Cancer Rep (Hoboken). 2026 Oct 25;9(10):e70707. doi: 10.1002/cnr2.70707 (PMC13696747; doi:10.1002/cnr2.70707)
Supplement: Supplementary file 4 [file CNR2-9-e70707-s005.docx]

**Supplementary Figure 2B. Time from initiation of first-line therapy to initiation of second-line therapy by ethnicity.** Kaplan–Meier curves comparing the time from initiation of first-line systemic therapy to initiation of second-line therapy between Hispanic American (HA; n = 15) and non-Hispanic White (NHW; n = 40) patients with metastatic hormone-sensitive prostate cancer who received second-line treatment. Patients who had not initiated second-line therapy by the time of last follow-up were censored. The median time from first-line to second-line therapy was **20.0 months** (95% confidence interval [CI], 8.0–23.0) for HA patients and **11.0 months** (95% CI, 9.0–13.0) for NHW patients. Survival curves were estimated using the Kaplan–Meier method and compared using the log-rank test (p = 0.0544). **Abbreviations:** HA, Hispanic American; NHW, non-Hispanic White; CI, confidence interval.

Log Rank Test=0.0544

| Time From First Line to Second Line by Ethnicity | | | | | |
| --- | --- | --- | --- | --- | --- |
|  | N | Median Time to First Line | Std. Error | 95% Confidence Interval | |
| NHW | 40 | 11.0 | 1.1 | 9.0 | 13.0 |
| HA | 15 | 20.0 | 3.9 | 8.0 | 23.0 |
